# The Pyrimidine Nucleotide Biosynthetic Pathway Modulates Production of Biofilm Determinants in *Escherichia coli*

**DOI:** 10.1371/journal.pone.0031252

**Published:** 2012-02-16

**Authors:** Marco Garavaglia, Elio Rossi, Paolo Landini

**Affiliations:** Department of Biomolecular Sciences and Biotechnology, Università degli Studi di Milano, Milan, Italy; University of Massachusetts Medical School, United States of America

## Abstract

Bacteria are often found in multicellular communities known as biofilms, which constitute a resistance form against environmental stresses. Extracellular adhesion and cell aggregation factors, responsible for bacterial biofilm formation and maintenance, are tightly regulated in response to physiological and environmental cues. We show that, in *Escherichia coli*, inactivation of genes belonging to the *de novo* uridine monophosphate (UMP) biosynthetic pathway impairs production of curli fibers and cellulose, important components of the bacterial biofilm matrix, by inhibiting transcription of the *csgDEFG* operon, thus preventing production of the biofilm master regulator CsgD protein. Supplementing growth media with exogenous uracil, which can be converted to UMP through the pyrimidine nucleotide salvage pathway, restores *csgDEFG* transcription and curli production. In addition, however, exogenous uracil triggers cellulose production, particularly in strains defective in either *carB* or *pyrB* genes, which encode enzymes catalyzing the first steps of *de novo* UMP biosynthesis. Our results indicate the existence of tight and complex links between pyrimidine metabolism and curli/cellulose production: transcription of the *csgDEFG* operon responds to pyrimidine nucleotide availability, while cellulose production is triggered by exogenous uracil in the absence of active *de novo* UMP biosynthesis. We speculate that perturbations in the UMP biosynthetic pathways allow the bacterial cell to sense signals such as starvation, nucleic acids degradation, and availability of exogenous pyrimidines, and to adapt the production of the extracellular matrix to the changing environmental conditions.

## Introduction

Bacteria are able to switch between two different “lifestyles”: single planktonic cells and sessile microbial communities, or biofilms. Biofilm cells are characterized by production of adhesion factors and extracellular polysaccharides (EPS) constituting the so-called “biofilm matrix” that, in addition to promoting cell-cell aggregation and cell-surface adhesion, can confer bacterial cell resistance to various environmental stresses [1]–[4]. Transition from planktonic cells to biofilm, as well as biofilm maturation and dispersal, responds to environmental and physiological cues, usually relayed to the bacterial cell by signal molecules. Accumulation of signal molecules triggers biofilm formation and maintenance by stimulating the production of adhesion factors, either by activating transcription of corresponding genes or by increasing activity of EPS biosynthetic enzymes. In Gram negative bacteria, the modified nucleotide cyclic-di-GMP (c-di-GMP) plays a pivotal role in biofilm formation and maintenance by stimulating production of EPS and adhesion factors [5]–[8], while negatively affecting cell motility [9], [10]. Another class of signal molecules, homoserine lactones, can promote biofilm formation in the opportunistic pathogen *Pseudomonas aeruginosa* by promoting production of biosurfactants [11], [12], and by stimulating production of extracellular DNA [13] and of lectins, proteins able to promote cell adhesion to sugar moieties [14]. In addition to dedicated signal molecules, intermediates and products of different metabolic pathways can also affect biofilm formation: for instance, indole, a product of tryptophan degradation, stimulates EPS production in *Vibrio cholerae* [15]. Likewise, glucose and glycolysis intermediates can greatly impact adhesion factors' production through different regulatory mechanisms (reviewed in [16]).

In *Escherichia coli* and other enterobacteria, curli amyloid fibers greatly enhance cell aggregation and adhesion to surfaces. Genes involved in curli biosynthesis are clustered in the *csgBAC* operon, encoding curli structural components, and the *csgDEFG* operon, encoding the CsgD transcription regulator and proteins involved in curli assembly and transport [17], [18]. The CsgD protein activates transcription of the *csgBAC* operon and of several genes involved in production of cell surface-associated structures and in cell adaptation to the biofilm lifestyle [19]–[21], including the *adrA* gene, encoding a diguanylate cyclase able to trigger cellulose production via c-di-GMP synthesis [6], [22]. Thus, curli, cellulose and other cell surface-associated structures are co-produced in a CsgD-dependent fashion to constitute the biofilm extracellular matrix. Expression of the *csg* operons takes place in response to a combination of environmental conditions: low growth temperature (<32°C), low osmolarity, and slow growth [18], and it is strongly dependent on the signal molecule c-di-GMP [7], [8]. A number of regulators, including OmpR, IHF, H-NS, CpxR, Crl, and the RpoS protein, play a role in curli gene expression [18], [23]–[25]. However, several aspects of curli regulation are still unclear: for instance, the molecular mechanisms of temperature dependence have not yet been fully elucidated, and no c-di-GMP sensor element involved in *csg* activation has been identified so far.

In this work, we show that curli and cellulose production are tightly linked to nucleotide biosynthetic pathways. In particular, transcription of the curli operons is strongly affected by pyrimidine nucleotide availability, while cellulose production is activated in the presence of exogenous uracil. Our observations suggest that production of cellulose and curli, usually co-regulated, can be unbalanced depending on the activity of different UMP biosynthetic pathway. Coupling of curli and cellulose production to UMP biosynthesis modulates formation of extracellular structures in response to physiological and environmental cues, such as starvation, nucleic acid turnover, and availability of exogenous pyrimidines.

## Results

### Mutations in the *carB* gene affect curli production

**Figure 1:**
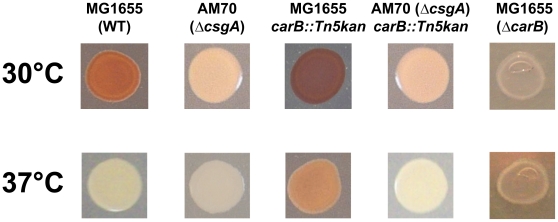
Determination of curli production by Congo red binding. Phenotypes on CR medium of MG1655 (wild type strain), AM70 (*csgA* deletion mutant, unable to produce curli), MG1655*carB::Tn5kan*, MG1655*carB::Tn5kan ΔcsgA::cat* and MG1655*ΔcarB::cat*. Strains were grown either at 30°C (for 24 hours) or at 37°C (for 18 hours). Plates were incubated for 48 hours at 4°C to enhance Congo red binding.

**Figure 2:**
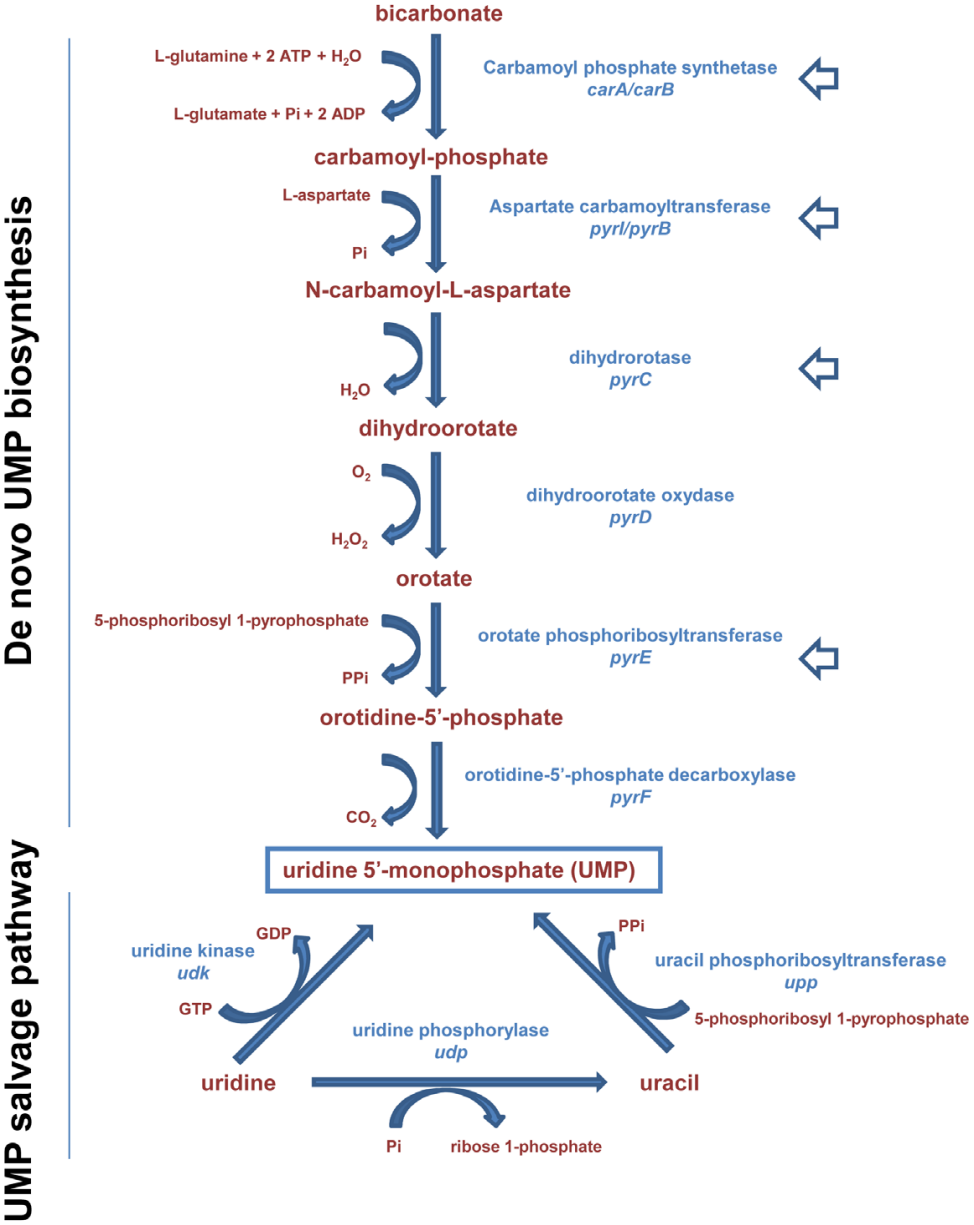
UMP biosynthetic pathways in *Escherichia coli*. Adapted from Ecocyc (http://ecocyc.org/).

Amyloid fibers such as curli bind to the dye Congo red very efficiently [17]; thus, phenotype on Congo red-supplemented agar medium (CR medium, see Materials and Methods) provides a convenient method for curli detection and an easy way to screen mutants affected in curli production (Figure 1). To identify novel genes involved in curli regulation, we carried out transposon mutagenesis in the *E. coli* strain MG1655; mutants were screened for their phenotype on CR medium both at 30°C and 37°C, *i.e.*, at permissive and non-permissive temperature for curli production. Several mutants were isolated that showed altered phenotype on CR medium (data not shown): one mutant displaying a dark red phenotype at 30°C and a weak red coloration at 37°C, suggesting increased curli production (Figure 1), was further characterized. Mapping of the Tn5<R6Kγori/KAN-2> transposon indicated that the insertion site lay in the *carB* gene, encoding a subunit of carbamoyl phosphate synthetase, which catalyzes the first step in the *de novo* pyrimidine nucleotide biosynthetic pathway (Figure 2). To verify that changes in phenotype in the *carB::Tn5kan* mutant of MG1655 were indeed due to altered curli production, we transduced the mutation in a strain unable to produce curli: the MG1655*carB::Tn5kan ΔcsgA::cat* double mutant displayed a white phenotype on CR medium both at 30°C and at 37°C (Figure 1), thus indicating that the dark red phenotype of the MG1655*carB::Tn5kan* mutant is totally dependent on curli fibers.

Several pieces of evidence indicated that the *carB::Tn5kan* mutation does not result in the inactivation of carbamoyl phosphate synthase activity: the MG1655*carB::Tn5kan* mutant was not auxotrophic for pyrimidines, nor did it show any defect in growth rate on minimal medium. Finally, its phenotype on CR medium was not reversed by complementation with the wild type *carB* allele on a multicopy plasmid (data not shown). The insertion site for the *Tn5kan* transposon occurs at nucleotide 2720 of the *carB* gene, corresponding to the 907^th^ codon, likely resulting in the production of a truncated form of the CarB protein lacking its regulatory domain involved in allosteric inhibition of protein activity by UMP [26]. Loss of the regulatory domain suggests that CarB protein activity might be increased in the MG1655*carB::Tn5kan* mutant strain. To verify this hypothesis, we constructed a *carB* mutant in which the portion of the gene encoding the catalytic domain of the CarB protein had been deleted (MG1655*ΔcarB::cat*). As expected, this mutant was auxotrophic for pyrimidines, and showed reduced growth rate in LB1/4 medium (data not shown). Addition of uracil at 0.25 mM to LB1/4 medium (LB1/4(ura)) fully overcame MG1655*ΔcarB::cat* partial growth defect (data not shown). The MG1655*ΔcarB::cat* mutant displayed a white phenotype on CR medium, suggesting inability to produce curli (Figure 1), thus confirming the hypothesis that the *carB::Tn5kan* mutation does indeed result in enhanced carbamoyl phosphate synthetase activity.

### Inactivation of UMP biosynthetic genes inhibits curli production at gene transcription level

**Figure 3:**
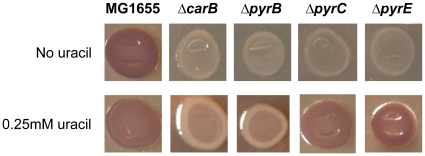
Congo red binding by *E. coli* strains deficient in UMP biosynthesis. The MG1655 strain and isogenic mutants deficient in UMP biosynthetic genes were spotted on either CR medium or CR(ura) medium (CR medium supplemented with 0.25 mM uracil) and grown for 24 hours at 30°C. Plates were incubated for 48 hours at 4°C to enhance Congo red binding.

To investigate whether the effects of *carB* inactivation could also be observed for other genes belonging to the *de novo* UMP biosynthetic pathway, we constructed knock out mutants in the *pyrB*, *pyrC* and *pyrE* genes, and tested them for their phenotypes on CR medium. As shown in Figure 3, inactivation of any UMP biosynthetic gene resulted in white phenotype on CR medium, indicating that curli production is inhibited by pyrimidine nucleotide starvation, rather than by lack (or accumulation) of any specific intermediate in the UMP biosynthetic pathway. Consistent with this result, strains impaired in *de novo* UMP biosynthesis were deficient in surface attachment experiments (Figure S1). To elucidate the mechanism of curli inhibition by perturbation of UMP biosynthesis, we measured transcript levels of the *csgD* and *csgB* genes, representatives of the two curli biosynthetic operons, using quantitative Real Time PCR (Table 1). Transcript levels of both *csgD* and *csgB* genes were dramatically decreased in every mutant deficient in UMP biosynthesis; in contrast, they were increased by approximately 3.5-fold in the MG1655*carB::Tn5kan*, in agreement with the dark red phenotype observed in this mutant (Figure 1). Consistent with inhibition of *csgDEFG* transcription, transcript levels of the CsgD-dependent *adrA* gene were also reduced by roughly 10-fold by mutations negatively affecting *de novo* UMP biosynthesis (Table 1).

**Table 1:** Determination of gene expression levels.

	LB1/4				LB1/4(ura)			
	*csgD*	*csgB*	*adrA*	*bcsA*	*csgD*	*csgB*	*adrA*	*bcsA*
MG1655	100*	100*	100*	100*	84.7	81.8	107	79.6
MG1655*carB::Tn5kan*	386	352	227	106	101	114	119	88.5
MG1655*ΔcarB::cat*	1.3	0.7	12.5	85.4	91.2	90.7	102	92.9
MG1655*ΔpyrB::cat*	0.7	0.1	n.d.	88.6	100.3	78.4	n.d.	102.5
MG1655*ΔpyrC::cat*	0.8	0.1	10.8	91.4	90.6	92.1	113	83.1
MG1655*ΔpyrE::cat*	0.5	0.2	n.d.	82.1	83.4	86.2	n.d.	86.4

Relative expression of the *csgD*, *csgB*, *adrA* and *bcsA* genes determined by Real-Time PCR on RNA extracted from overnight cultures. 16S RNA transcript was used as reference gene. *Δ*Ct values between the genes of interest and 16S RNA were set at 100 for MG1655 in LB1/4 medium, and transcript levels in other strains and/or growth conditions are expressed as relative values. Experiments were repeated at least three times, each time in duplicate; standard deviations were always lower than 5%.

In contrast to the genes belonging to the CsgD regulon, relative amounts of 16S rRNA, used as reference gene in Real Time PCR experiments, were similar in MG1655 and in the strains carrying non-functional alleles of UMP biosynthetic genes (data not shown), as were transcript levels of the cellulose biosynthetic *bcsA* gene, which is not regulated by the CsgD protein [27], [28] (Table 1). These results strongly suggest that pyrimidine starvation leads to a reduction in *csgD* and *csgB* transcript levels via a specific mechanism rather than through a general inhibition of transcription. To determine whether reduction in *csgD* transcript levels could depend on decreased mRNA stability, we performed mRNA decay experiments, which did not show any significant difference in *csgD* mRNA half-lives in MG1655*ΔcarB::cat* in comparison to MG1655 (data not shown), suggesting that knock out mutations in the *de novo* UMP biosynthetic pathway affects *csgD* expression at the transcription initiation step.

Upon addition of 0.25 mM uracil to LB1/4 medium (LB1/4(ura) medium) transcription of both *csgD* and *csgB* was re-established in mutant strains affected in *de novo* UMP biosynthesis (Table 1), thus confirming that *csgDEFG* expression is repressed by pyrimidine starvation. However, surprisingly, addition of uracil to CR medium (CR(ura) medium) failed to restore the curli-dependent red phenotype in the *carB* and *pyrB* strains (Figure 3), in apparent contradiction with the results of the gene expression experiments. In contrast, the MG1655 strain, as well as the MG1655*ΔpyrC* and MG1655*ΔpyrE* mutants, displayed a red phenotype on CR(ura) medium, which was not affected by supplementing uracil up to a final concentration of 1 mM (data not shown). Surface adhesion experiments showed that growth in LB1/4(ura) only partially restored ability to form biofilm in the MG1655*ΔcarB::cat* and MG1655*ΔpyrB::cat* strains (Figure S1). These results could suggest that, although curli operon transcription was fully resumed in the presence of additional uracil, curli subunit production might still be impaired in the MG1655*ΔcarB::cat* and MG1655*ΔpyrB::cat* strains. However, determination of curli fibers' production using the SDS-agarose electrophoresis method [29] performed on MG1655*ΔcarB::cat* showed that was fully competent for curli production when grown in LB1/4(ura) solid medium (Figure S2), in agreement with gene expression experiments (Table 1).

### Effects of regulatory proteins affecting pyrimidine metabolism and of inhibition of purine biosynthesis on curli production

**Figure 4:**
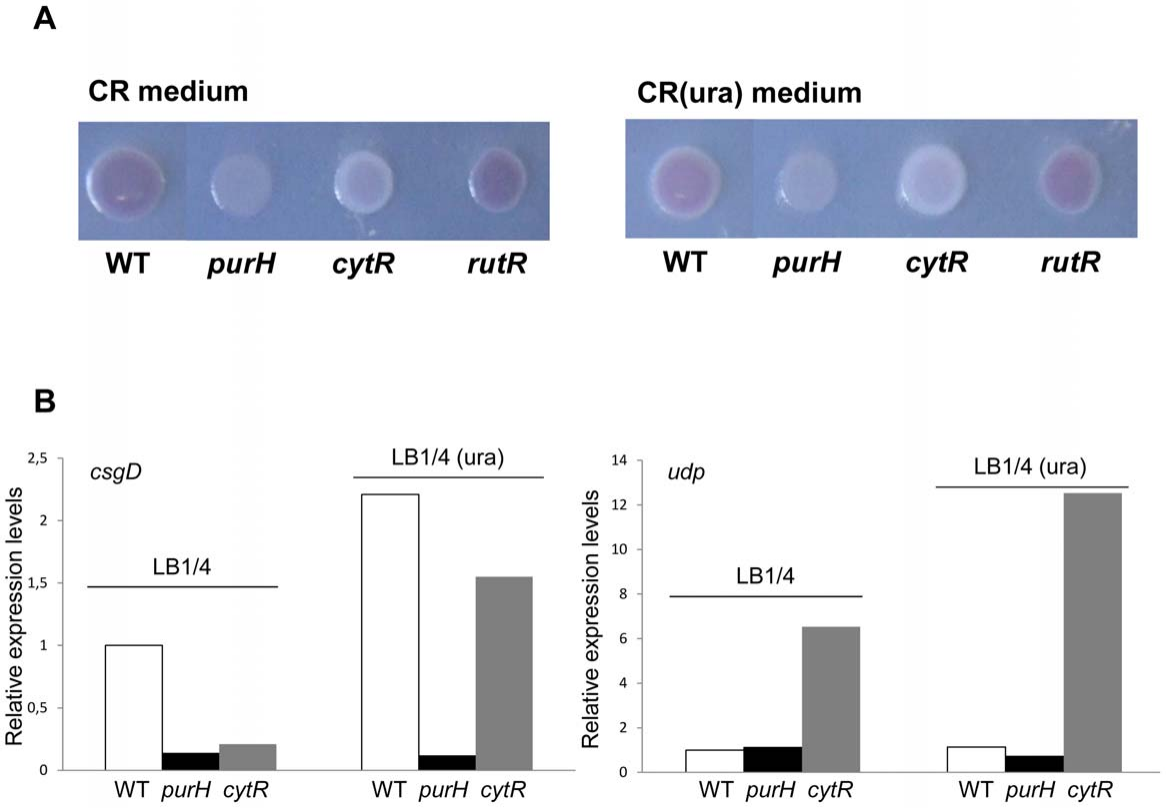
Congo red binding by *E. coli* strains deficient in pyrimidine sensing (*cytR* and *rutR* mutants) and purine biosynthesis (*purH* mutant). **4A.** The MG1655 strain and its isogenic mutants in the *purH*, *cytR* and *rutR* genes were spotted either on CR medium (left panel) or on CR(ura) medium (right panel) and grown for 24 hours at 30°C. Plates were incubated for 48 hours at 4°C to enhance Congo red binding. Determination of transcript levels. **4B.** Relative expression of either the *csgD* gene (left panel) or the *udp* gene (right panel) was determined by Real-Time PCR on RNA extracted from overnight cultures of MG1655 and of its isogenic *purH* and *cytR* mutants. 16S RNA transcript was used as reference gene. *Δ*Ct values between the genes of interest and 16S RNA were set at 1 for MG1655 in LB1/4 medium, and transcript levels in other strains and/or growth conditions are expressed as relative values. Experiments were repeated at least three times, each time in duplicate; standard deviations were always lower than 5%.

We investigated whether pyrimidine starvation might affect curli production and *csg* gene expression via known pyrimidine-sensing regulatory proteins. To this aim, we constructed isogenic mutants of MG1655 in which either the *cytR* or the *rutR* gene were inactivated. The CytR protein is a repressor of genes involved in pyrimidine uptake and degradation; negative regulation by CytR is relieved by high intracellular concentrations of cytidine [30]. Interestingly, in *Vibrio cholerae*, a CytR-like protein negatively controls biofilm formation by repressing EPS production [31]. DNA binding by RutR, a regulator of genes involved both in pyrimidine biosynthesis and degradation, is inhibited by uracil [32]. Thus, both CytR and RutR proteins regulate gene expression in response to intracellular pyrimidine concentrations. We tested the effects of the *cytR* and of the *rutR* mutations on CR phenotype, either in the presence or in the absence of exogenous uracil (Figure 4A): inactivation of the *rutR* gene did not affect CR phenotype, while, in contrast, the MG1655*ΔcytR* mutant strain displayed a white phenotype both on CR and on CR(ura) medium, indicative of reduced curli production. *csgD* transcript levels are reduced by roughly 5-fold in the *ΔcytR* mutant strain grown in LB1/4 medium, but they are restored to wild type levels by addition of 0.25 mM uracil (Figure 4B). In contrast, expression of the CytR-dependent *udp* gene, used as a control in gene expression experiments, are increased in the *cytR* mutant regardless of the presence of exogenous uracil, as expected (Figure 4B). Thus, the behavior of the *cytR* mutant with respect to curli production and *csgD* gene expression strongly resembles the MG1655*ΔcarB::cat* and MG1655*ΔpyrB::cat* strains (see Figure 3 and Table 1). These observations suggest that the CytR protein does not mediate pyrimidine-dependent regulation of the *csg* operons directly; however, lack of a functional *cytR* gene likely results in altered intracellular pyrimidine concentrations, which would in turn affect *csgDEFG* expression and curli production.

Our results show that pyrimidine starvation-dependent downregulation of *csgDEFG* expression and of curli production is not mediated by regulatory proteins directly involved in sensing intracellular pyrimidine concentrations. Thus, we hypothesized that pyrimidine starvation might downregulate *csgDEFG* expression through a general effect on intracellular nucleotide pools. As an initial verification of this hypothesis, we tested the effects of purine starvation on curli production and *csgDEFG* expression. Inactivation of the purine biosynthetic gene *purH* resulted in white phenotype on CR medium (Figure 4A) and in a 7-fold reduction of *csgDEFG* transcript levels (Figure 4B). Similar to what observed for mutations in *de novo* pyrimidine biosynthesis, *purH* inactivation does not result in a non-specific downregulation of transcription, since transcript levels of the CsgD-independent *udp* gene were unaffected in the *purH* mutant strain (Figure 4B). As expected, addition of 0.25 mM uracil did not revert the effects of the *purH* mutation (Figure 4), indicating that uracil can only counteract the effects of mutations specifically affecting UMP concentrations.

Since curli production and *csgDEFG* expression are strongly dependent upon the signal molecule c-di-GMP [7], [8], it is conceivable that changes in the nucleotide pools due to mutation in nucleotide biosynthetic genes could affect c-di-GMP production. This would be in agreement with our previous observations that sulfathiazole, a sulfonamide drug interfering with nucleotide biosynthesis, can inhibit c-di-GMP biosynthesis [33]. Determination of intracellular c-di-GMP concentrations did not show significant differences in MG1655*ΔcarB::cat* and the MG1655*carB::Tn5kan* strains in comparison to MG1655 (data not shown); however, c-di-GMP concentrations in MG1655 cells are in the nanomolar range [33], making a precise determination of c-di-GMP in cell extracts rather difficult. In addition, it must be pointed out that induction of one specific diguanylate cyclase, sufficient for activation of its corresponding target, might not result in any significant increase in the overall concentration of intracellular c-di-GMP.

### Uracil triggers cellulose production

**Figure 5:**
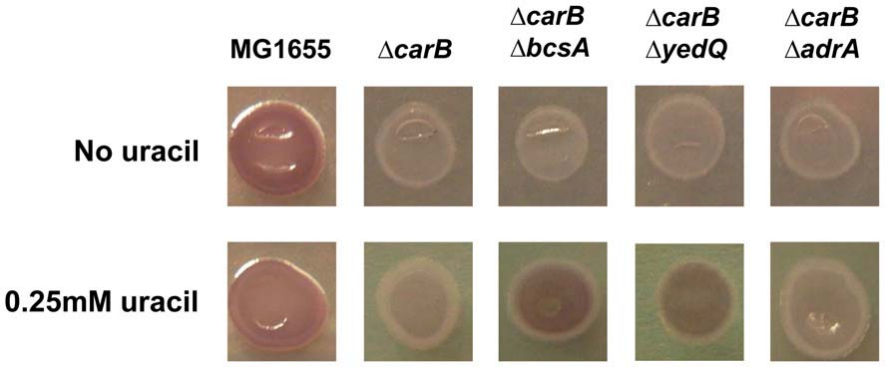
Effect of cellulose production on Congo red binding. Phenotypes on CR medium of MG1655, MG1655*ΔcarB::cat*, MG1655*ΔcarB::cat ΔbcsA::kan*, MG1655*ΔcarB::cat ΔyedQ::kan*, MG1655*ΔcarB::cat ΔadrA::kan*. Strains were spotted on either CR medium or CR(ura) medium (CR medium supplemented with 0.25 mM uracil) and grown for 24 hours at 30°C. Plates were incubated for 48 hours at 4°C to enhance Congo red binding.

**Figure 6:**
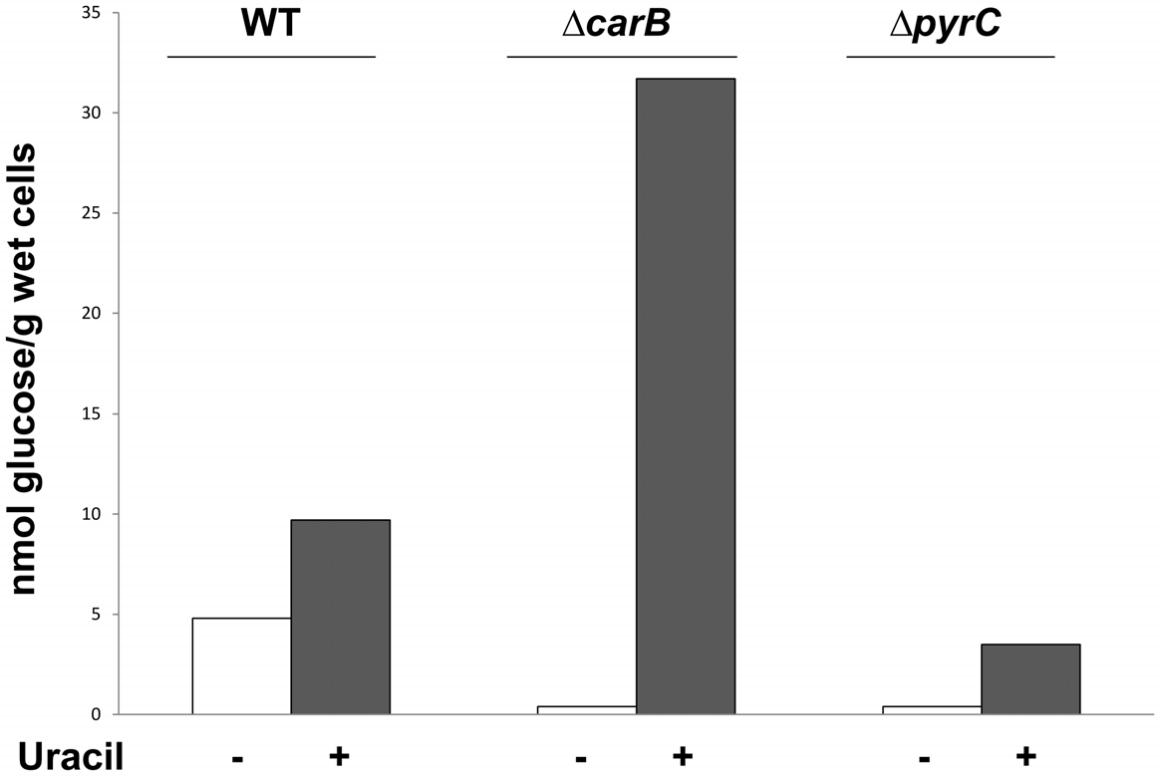
Determination of cellulose amounts. Strains MG1655, MG1655*ΔcarB::cat*, and MG1655*ΔpyrC::tet* were grown 48 hours at 30°C on either LB1/4 agar (no added uracil, Cellulose extraction and determination was performed as described [35]. Data shown are the average of two independent experiments giving very similar results. For strains MG1655*ΔcarB::cat* and MG1655*ΔpyrC::tet* grown on LB1/4 agar no glucose was detectable in the assays. A value of 0.5 nmol glucose, corresponding to the lowest detectable concentration in the assay, as determined by a glucose standard curve, was thus arbitrarily assigned to these strains.

Results presented in this work (Figure 3, Table 1, Figure S1) suggest that, in the MG1655*ΔcarB::cat* and MG1655*ΔpyrB::cat* strains grown in LB1/4(ura) medium, exposure of curli fibers on the cell surface might be hindered by production of additional extracellular structures. Indeed, it is known that overproduction of cellulose and other EPS can prevent curli-mediated Congo red binding and cell adhesion [34]–[37]. To test the possibility that exogenous uracil might affect phenotypes on CR medium in the MG1655*ΔcarB::cat* and the MG1655*ΔpyrB::cat* strains via cellulose overproduction, we inactivated *bcsA*, the first gene of the cellulose biosynthetic operon, in these genetic backgrounds. Deletion of the *bcsA* gene restored, albeit partially, the red phenotype on CR(ura) medium (Figure 5, data not shown), suggesting that the white phenotype on CR(ura) medium might indeed depend on EPS overproduction. Likewise, it resulted in efficient surface attachment by the MG1655*ΔcarB::cat ΔbcsA::kan* double mutant (Figure S1). In contrast, deletion of the *bcsA* gene did not affect either CR phenotype or surface attachment in the MG1655 strain (data not shown), in agreement with previous observations [35]. To confirm our hypothesis further, we determined cellulose amounts in the MG1655, MG1655*ΔcarB::cat* and MG1655*ΔpyrC::tet* strains grown either in LB1/4 or in LB1/4(ura). Although growth in LB1/4(ura) enhanced cellulose production in all strains tested, this effect was much stronger in MG1655*ΔcarB::cat*, leading to production of a 3.5-fold higher amount of cellulose in comparison to MG1655 grown in the same conditions (Figure 6).

Enzymatic activity of the cellulose biosynthetic machinery is subject to regulation by the signal molecule c-di-GMP. Two distinct c-di-GMP synthetases, the AdrA and YedQ proteins, can activate cellulose production; although AdrA overexpression has been reported to affect curli production [7], [35], in physiological conditions AdrA sole function is to activate cellulose production [27], [37]. AdrA and YedQ act independently and belong to two distinct regulatory circuits [6], [27], [38]: indeed, while AdrA is encoded by a CsgD-dependent gene [22], thus presiding to co-ordinated production of curli and cellulose, YedQ expression and activity are independent of CsgD [38], [39]. We tested the hypothesis that exogenous uracil might affect cellulose production via c-di-GMP synthesis by either AdrA or YedQ. Interestingly, inactivation of the *yedQ* gene, but not of *adrA*, in either the MG1655*ΔcarB::cat* (Figure 5) or the MG1655*ΔpyrB::cat* (data not shown) genetic backgrounds partially restored red phenotypes on CR medium, similar to the MG1655*ΔcarB::cat ΔbcsA* double mutant, thus suggesting that cellulose overproduction in the presence of exogenous uracil is mediated by the YedQ protein.

## Discussion

In this work, we have shown that mutations in genes belonging to *de novo* nucleotide biosynthetic pathways strongly affect *csgDEFG* expression and curli production in *E. coli* (Figure 1, Figures 3–4, Table 1, Figures S1, S2). Interplay between nucleotide metabolism and biofilm appears to be conserved in different bacteria; however, specific effects and mechanism may vary substantially. Indeed, although our results are consistent with previous findings showing that active *de novo* UMP biosynthesis is necessary for biofilm formation in *P. aeruginosa* [40], [41], in this bacterium inhibition of purine biosynthesis through inactivation of the *purH* gene does not affect adhesion factors' production [41], in contrast to what observed in *E. coli* (Figure 4). Likewise, pyrimidines appear to control EPS production and biofilm formation in *V. cholerae* through the dedicated regulator CytR [31], which does not appear to play a direct role in curli regulation in *E. coli* (Figure 4). Despite these differences, it seems that absence of *de novo* pyrimidine biosynthesis can act as a signal for severe nutrient starvation, which can in turn prevent biofilm formation and promote biofilm dispersal [42].

In *E. coli*, the effects of mutations in the *de novo* UMP biosynthesis on curli production can be complemented by supplementing growth medium with uracil, thus suggesting that pyrimidine nucleotide availability, regardless whether it is achieved via *de novo* UMP biosynthesis or the pyrimidine salvage pathway, allows efficient *csgDEFG* transcription and expression of the CsgD regulon (Table 1, Figure S2). Regulation of *csgDEFG* expression by intracellular nucleotide concentrations might take place by direct modulation of transcription initiation by RNA polymerase, similar to transcription control by GTP availability described for ribosomal promoters [43], or through not yet identified nucleotide-sensing regulatory proteins. Alternatively, perturbations in nucleotide pools might affect accumulation of c-di-GMP, a signal molecule necessary for *csgDEFG* expression [7], [8], possibly by impairing diguanylate cyclases' enzymatic activity.

Diguanylate cyclases play a role in pyrimidine-dependent regulation of cellulose production. Cellulose production is regulated by a more complex mechanism since, in addition to pyrimidine availability, it seems to respond to the relative activity of the two UMP biosynthetic pathways. Indeed, MG1655 produces twice as much cellulose when grown in the presence of exogenous uracil (Figure 6), *i.e.*, in conditions in which UMP biosynthesis is mostly carried out via the pyrimidine salvage pathway and *de novo* UMP biosynthesis is inhibited [32], [44]. Induction of cellulose production by exogenous uracil is further enhanced in mutants carrying non-functional *carB* or *pyrB* alleles (Figures 3, 5–6 and data not shown): in contrast, strains carrying mutations in later steps of the *de novo* UMP biosynthetic pathway, such as MG1655*ΔpyrC::tet*, do not overproduce cellulose in response to uracil (Figure 3, Figure 6). These observations suggest that bacterial cells might sense the molecular ratio between UMP and intermediates in the *de novo* UMP biosynthesis such as carbamoyl-L-aspartate, which accumulates in the *pyrC* mutant strain, as a signal of the relative balance between the two UMP biosynthetic pathways. An unbalance towards UMP biosynthesis via the pyrimidine salvage pathway triggers cellulose production, and this effect relies on the activity of the diguanylate cyclase YedQ (Figure 5).

The interplay between nucleotide salvage pathway and cellulose production might be connected to the role of cellulose and other EPS in the response to environmental stresses such as desiccation and resistance to bacteriophages [3], [35], [45]. In bacterial biofilms, events leading to extensive cell lysis, such as exposure to antibiotics or attack by bacteriophages, would release cell components into the local environment: thus, a sudden increase in concentrations of exogenous nucleotides due to bacterial lysis might function as an “alarm signal” to neighboring cells, which would react by producing EPS as a defense mechanism against environmental stresses. For intracellular pathogenic enterobacteria, sensing an increase of exogenous nucleotide concentration might instead signal stress events in the host cell, such as leakage of nucleotides from the nuclear compartment. Consistent with our observations, it has been reported that allosteric inhibition of the CarB protein by exogenous uracil strongly influences production of extracellular structures and negatively affects expression of type III secretion systems in the intracellular pathogen *Shigella flexneri* [46]. In *Pseudomonas fluorescens*, a spontaneous mutation in the *carB* gene affects the proportion of capsulated and non-capsulated subpopulations via yet unknown molecular mechanisms [47]. Our results complement and expand these observations, and underline the importance of the interplay linking biofilm formation, bacterial virulence, production of extracellular structures, and nucleotide biosynthetic pathways: better understanding of these connections at the molecular level will allow us to improve our strategies in preventing (or promoting) bacterial biofilms. In this perspective, our results provide strong evidence to confirm previous findings suggesting that drugs targeting nucleotide biosynthetic pathways have a strong potential as antibiofilm agents [33], [41].

## Materials and Methods

### Bacterial strains and growth conditions

Bacterial strains used in this work are listed in Table 2. For strain construction and manipulation, bacteria were grown in LB medium (10 g/L Tryptone, 5 g/L Yeast Extract, 5 g/L NaCl). For adhesion assays and gene expression regulation studies, bacteria were grown in LB medium diluted 1∶4 in H_2_O (LB1/4). The LB1/4 medium was used since it allows efficient induction of the CsgD regulon [48] and provides sufficient pyrimidines to partially overcome the growth defect of strains mutated in *de novo* UMP biosynthetic pathway. When required, LB1/4 medium was supplemented with 0.25 mM uracil (LB1/4(ura) medium); uracil was dissolved in 50% dimethyl sulfoxide (DMSO) in water at a concentration of 10 mM. DMSO to a 1.25% final concentration was always added to control cultures.

**Table 2:** *Escherichia coli* strains and plasmids used in this work.

*Escherichia coli*Strains	Relevant genotype or characteristics	Reference or source
MG1655	K-12, F^−^ λ^−^ *rph-1*	Standard laboratory strain [52]
AM70	MG1655 *ΔcsgA::cat*	[37]
LG28	MG1655 *ΔbcsA::kan*	[35]
LG30	MG1655 *ΔadrA::kan* obtained by P1 transduction from 3934*adrA* [38]	This work
MG1655*carB::Tn5kan*	*Tn5::kan* transposon inserted at nucleotide 2720 of the *carB* gene	This work
MG1655*ΔcarB::cat*	Replacement of the nucleotides 1–550 of the *carB* gene with a chloramphenicol resistance cassette	This work
MG1655*ΔcytR::cat*	Replacement of the *cytR* gene with a chloramphenicol resistance cassette	This work
MG1655*ΔpurH::cat*	Replacement of the *purH* gene with a chloramphenicol resistance cassette	This work
MG1655*ΔpyrB::cat*	Replacement of the *pyrB* gene with a chloramphenicol resistance cassette	This work
MG1655*ΔpyrC::tet*	Replacement of the *pyrC* gene with a tetracycline resistance cassette	This work
MG1655*ΔpyrE::tet*	Replacement of the *pyrE* gene with a tetracycline resistance cassette	This work
MG1655*ΔrutR::cat*	Replacement of the *rutR* gene with a chloramphenicol resistance cassette	This work
MG1655*ΔyedQ::kan*	Replacement of the *yedQ* gene with a kanamycin cassette	This work
MG1655*carB::Tn5kan ΔcsgA::cat*	Obtained by P1 transduction from AM70 into MG1655*carB::Tn5kan*	This work
MG1655*ΔcarB::cat ΔbcsA::kan*	Obtained by P1 transduction from LG28 into MG1655*ΔcarB::cat*	This work
MG1655*ΔcarB::cat ΔadrA::kan*	Obtained by P1 transduction from LG30 into MG1655*ΔcarB::cat*	This work
MG1655*ΔcarB::cat ΔyedQ::kan*	Obtained by inactivation of the *yedQ* gene by λ red technique	This work
MG1655*ΔpyrB::cat ΔbcsA::kan*	Obtained by P1 transduction from LG28 into MG1655*ΔpyrB::cat*	This work
MG1655*ΔpyrB::cat ΔadrA::kan*	Obtained by P1 transduction from LG30 into MG1655*ΔpyrB::cat*	This work
MG1655*ΔpyrB::cat ΔyedQ::kan*	Obtained by inactivation of the *yedQ* gene by λ red technique	This work
**Plasmids**		
pCR2.1	Control vector allowing direct cloning of PCR products, ampicillin resistance	Invitrogen
pCR2.1-*carB*	*carB* gene cloned as PCR product into pCR2.1 vector	This work

For Congo red (CR) binding assays, overnight cultures were spotted, using a replicator, on LB1/4 agar medium to which 0.004% Congo red and 0.002% Coomassie blue were added after autoclaving (CR medium). Bacteria were grown for 20 h at 30°C; phenotypes were better detectable after 24–48 h incubation at 4°C. When needed, antibiotics were used at the following concentrations: ampicillin, 100 µg/ml; chloramphenicol, 50 µg/ml; kanamycin, 50 µg/ml; tetracycline, 25 µg/ml; rifampicin, 100 µg/ml.

### Genetic techniques

Transposon insertion mutagenesis was carried out using the EZ-Tn5<R6Kγori/KAN-2> transposome (Epicentre). Transposon mutagenesis and determination of transposon insertion site by rescue cloning were carried out according to the manufacturer's instructions. *E. coli* MG1655 mutant derivatives were constructed either using the λ Red technique [49] or by bacteriophage P1 transduction [50]. The list of primers used for gene inactivation and for confirmation of target gene disruption by PCR is presented in Table S1. Construction of the pCR2.1-*carB* plasmid was carried out by PCR amplification of the *carB* gene from the MG1655 genome followed by direct cloning of the PCR product into the pCR2.1vector (Invitrogen).

### Gene expression studies

Determination of relative gene expression levels was performed by quantitative Real Time PCR, using bacterial cultures grown either in LB1/4 or in LB1/4(ura) at 30°C, and harvested either from overnight cultures or from exponential phase (OD_600 nm_ = 0.6 for MG1655 and MG1655*carB::Tn5kan*, OD_600 nm_ = 0.2 for strains carrying null mutations in UMP biosynthetic genes). Primers for Real-Time PCR are listed in Table S1. mRNA stability was measured by Real-Time PCR experiments in the presence of rifampicin as described [51]. 16S RNA was always used as reference gene.

### Other methods

Detection of curli amyloid fibers was performed using the SDS-agarose electrophoresis method as described [29]. Cellulose amount was estimated on bacterial cultures grown on solid medium for 48 hours; cells were collected, resuspended in H_2_O and centrifuged at 12.000×g for 10 minutes; cellulose was determined as glucose released from cellulase treatment on culture supernatants as previously described [35]. Biofilm formation was determined with the surface attachment assay in microtiter plates [35] performed on bacterial cultures grown overnight in LB1/4 at 30°C.

## Supporting Information

Figure S1 **Surface adhesion on polystyrene microtiter plates.** Surface adhesion experiments were performed as previously described [35]. White bars: overnight cultures grown in LB1/4 medium; grey bars: overnight cultures grown in LB1/4(ura) medium. Three independent experiments were performed and standard deviations are shown.
(TIF)

Figure S2 **SDS-agarose gel.** Curli production was detected using the SDS-agarose gel method [29]. The same amount of total protein was loaded in each sample. Insoluble material, mostly constituted by curli amyloids, cannot migrate into the agarose gel and is stained by Coomassie blue. Cultures were grown on solid medium (LB1/4 agar or LB1/4(ura) agar) for 24 hours at 30°C.
(TIF)

Table S1 Primers used in this work.
(DOC)
