# Supplementary figures and images for: The Pyrimidine Nucleotide Biosynthetic Pathway Modulates Production of Biofilm Determinants in Escherichia coli
Source: PLoS One. 2012 Feb 16;7(2):e31252. doi: 10.1371/journal.pone.0031252 (PMC3281075; doi:10.1371/journal.pone.0031252)

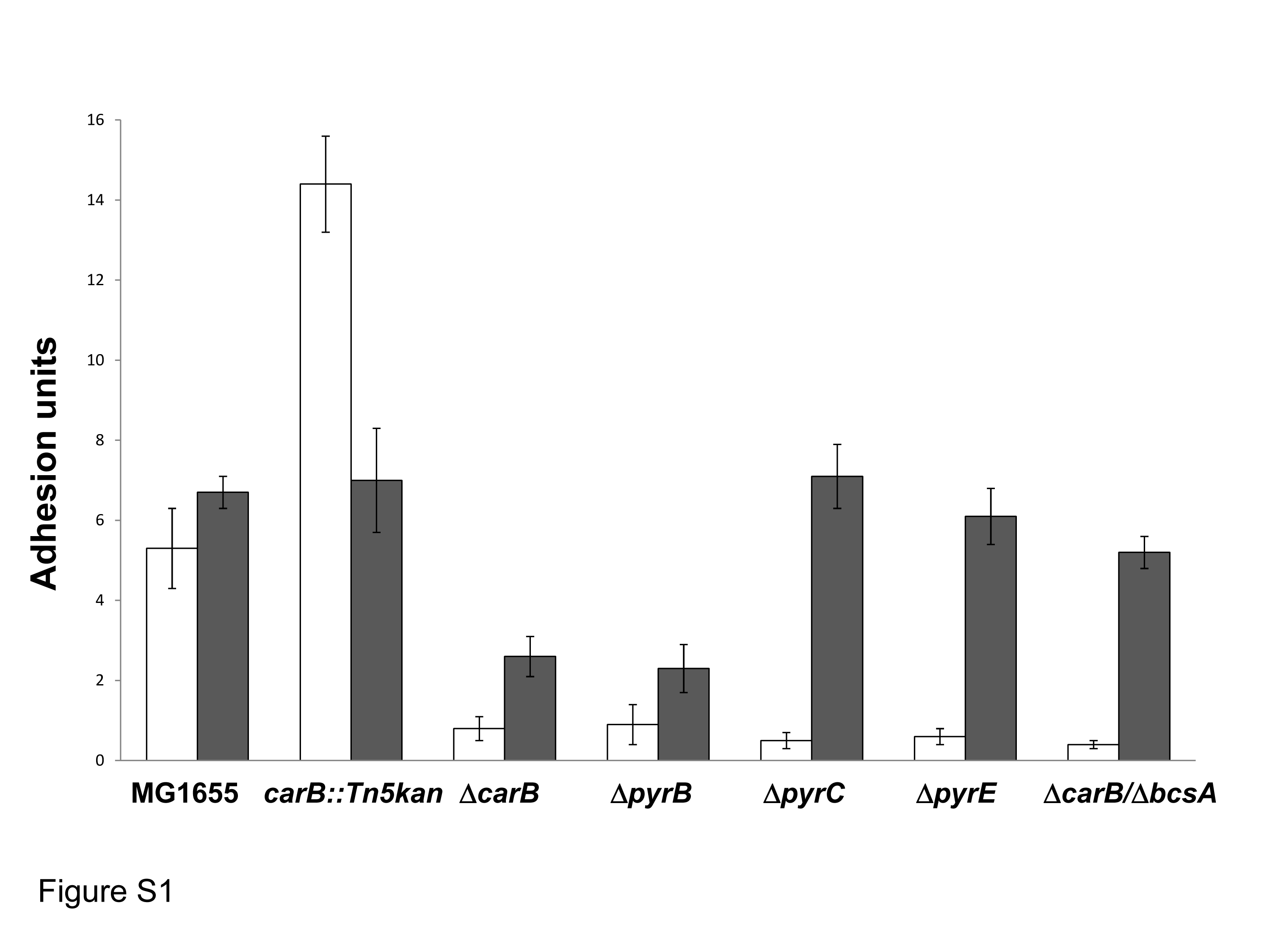

Supplement: Figure S1 — Surface adhesion on polystyrene microtiter plates. Surface adhesion experiments were performed as previously described [35]. White bars: overnight cultures grown in LB1/4 medium; grey bars: overnight cultures grown in LB1/4(ura) medium. Three independent experiments were performed and standard deviations are shown. (TIF) [file pone.0031252.s001.tif]

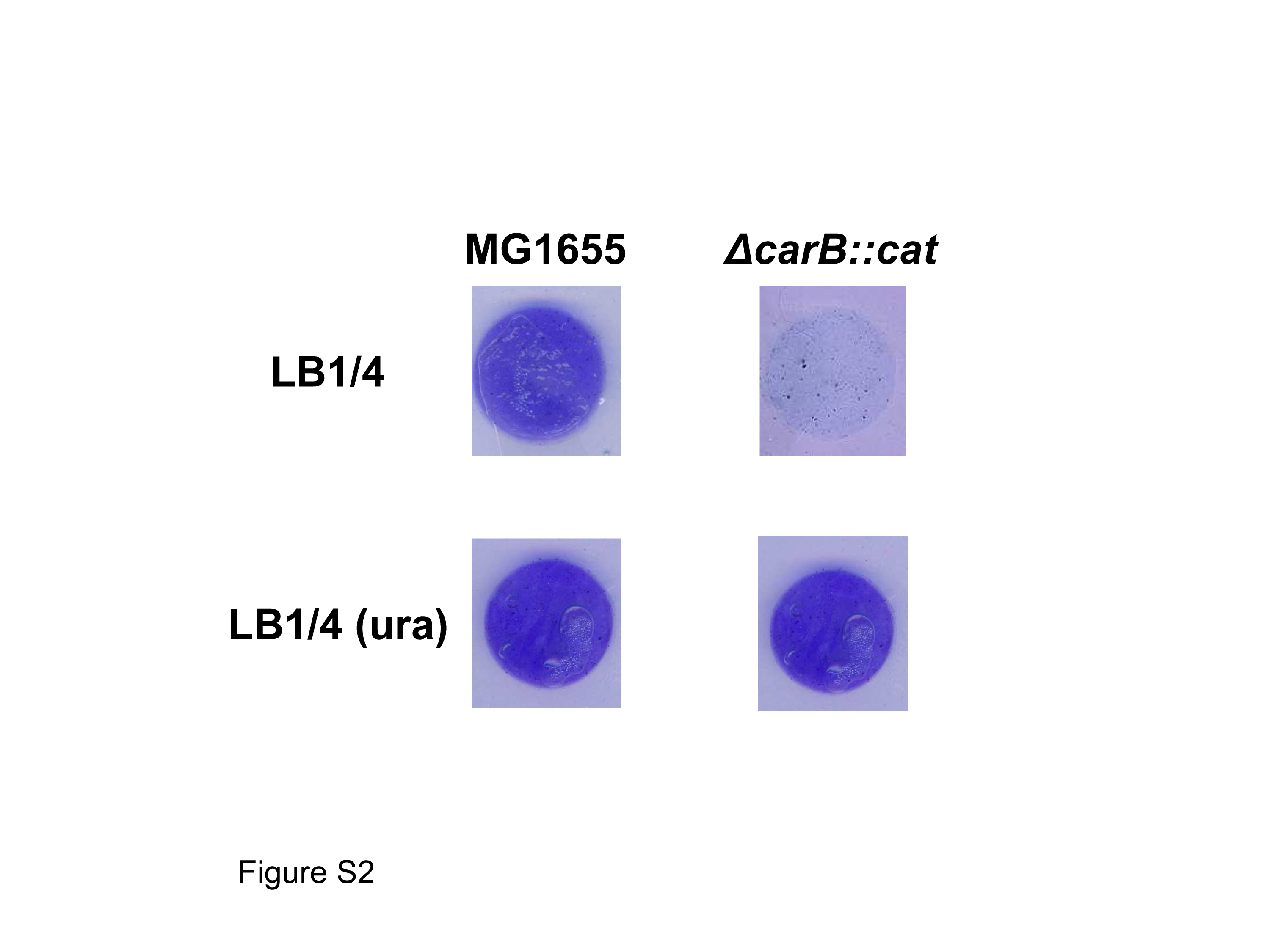

Supplement: Figure S2 — SDS-agarose gel. Curli production was detected using the SDS-agarose gel method [29]. The same amount of total protein was loaded in each sample. Insoluble material, mostly constituted by curli amyloids, cannot migrate into the agarose gel and is stained by Coomassie blue. Cultures were grown on solid medium (LB1/4 agar or LB1/4(ura) agar) for 24 hours at 30°C. (TIF) [file pone.0031252.s002.tif]
