# Supplementary material for: The Pyrimidine Nucleotide Biosynthetic Pathway Modulates Production of Biofilm Determinants in Escherichia coli
Source: PLoS One. 2012 Feb 16;7(2):e31252. doi: 10.1371/journal.pone.0031252 (PMC3281075; doi:10.1371/journal.pone.0031252)
Supplement: Table S1 — Primers used in this work. (DOC) [file pone.0031252.s003.doc]

| **Primers** | **Sequence** | **Utilization** |
| --- | --- | --- |
| carB_for | 5’-CAGTACCGTAAAACCGCT-3’ | *carB* cloning and mutant verification |
| carB_rev | 5’-ATATCTGCCATGACACGC-3’ | *carB* cloning |
| carB_cat_for | 5’-AGTACCGTAAAACCGCTAAGTAATCAGGAGTAAAAGAGCCTACCTGTGACGGAAGATCAC-3’ | *carB* inactivation |
| carB_cat_rev | 5’-AGCCCACGTCAGCGGCAACCGCCAGCGCTTCTTCCATCGTGGGCACCAATAACTGCCTTA-3’ | *carB* inactivation |
| pyrB_cat_for | 5’- TCGAGGGGCTTTTTTTTGCCCAGGCGTCAGGAGATAAAAGTACCTGTGACGGAAGATCAC-3’ | *pyrB* inactivation |
| pyrB_cat_rev | 5’- TCATCGAGCATATCCAGAATGTATTGCGGCATTGCCAGCGGGGCACCAATAACTGCCTTA-3’ | *pyrB* inactivation |
| pyrC_tet_for | 5’-GTCCGGCAAAAACATCCCTTCAGCCGGAGCATAGAGATTACTAGACATCATTAATTCCTA-3’ | *pyrC* inactivation |
| pyrC_tet_rev | 5’-CAGGTAAAATAACCTAATGACAACAGGAAGCTACGATTTGAAGCTAAATCTTCTTTATCG-3’ | *pyrC* inactivation |
| pyrE_tet_for | 5’-TTTTTTTTGTCTGTAGAAAAGTAAGATGAGGAGCGAAGGCCTAGACATCATTAATTCCTA-3’ | *pyrE* inactivation |
| pyrE_tet_rev | 5’-TAATATGACGCCGGATGACTTTTCATCCGGCGAGTTTCTGAAGCTAAATCTTCTTTATCG-3’ | *pyrE* inactivation |
| yedQ_FRT_for | 5’- GCCAGAATCATAAAAAAGCAGGTTGGGAGTCGTCAGGGTGGTGTAGGCTGGAGCTGCTTC-3’ | *yedQ* inactivation |
| yedQ_FRT_rev | 5’- GGCTGGACCATTTTTTCTCCGCCCGTTAAGCGTTATCGCTTTCCGGGGATCCGTCGACCT-3’ | *yedQ* inactivation |
| purH_FRT_for | 5’- AACGCTCTCTGTAATAGTCAAATCCAGGGGATTTACCATGGTGTAGGCTGGAGCTGCTTC-3’ | *purH* inactivation |
| purH_FRT_rev | 5’-ACTAATACTTTCATCTATTGCTCCATTAATGGCGGAAGTGTTCCGGGGATCCGTCGACCT-3’ | *purH* inactivation |
| cytR_FRT_for | 5’-GATGTAGTACGCCTGACGTGCCAGGCGAGGAGTGAGTGTGGTGTAGGCTGGAGCTGCTTC-3’ | *cytR* inactivation |
| cytR_FRT_rev | 5’-GTCACGGCAGTCTTAAAGGTTTACTTTAAGGTAACGCGCGTTCCGGGGATCCGTCGACCT-3’ | *cytR* inactivation |
| rutR_FRT_for | 5’-TGCTATCCTGTTGCCAATCTACAAGAGGGGAGAGCGCATGGTGTAGGCTGGAGCTGCTTC-3’ | *rutR* inactivation |
| rutR_FRT_rev | 5’-GATGTTACAACCTCCTCCGGCATCTTTAACGTGGTCGAATTTCCGGGGATCCGTCGACCT-3’ | *rutR* inactivation |
| pyrB_for | 5’-CTTCCCGTTGATCACCCATT-3’ | Mutant verification |
| pyrC_for | 5’-ATTTTCGTGCAAAGGAAAA-3’ | Mutant verification |
| pyrE_for | 5’-ATTTTTAAGGCGACTGAT-3’ | Mutant verification |
| yedQ_for | 5’-CGCTGTTTTGCGGTACGCTA-3’ | Mutant verification |
| purH_for | 5’-GCCCACGGTAACCACAGTCA-3’ | Mutant verification |
| cytR_for | 5’-GAGGGTTAAACCGCTCACGA-3’ | Mutant verification |
| rutR_for | 5’-GCGATGAGAGTGCAGAAGGT-3’ | Mutant verification |
| csgA_for | 5’-ACAGTCGCAAATGGCTATTC-3’ | Mutant verification |
| bcsA_for | 5’-CTAAGCAACCAGTAGGTGAATATC-3’ | Mutant verification |
| adrA_for | 5’-GCTCCGTCTCTATAATTTGGG-3’ | Mutant verification |
| cat_rev | 5’-GGGCACCAATAACTGCCTTA-3’ | Mutant verification |
| tet_rev | 5’-TGCAGGTAAAGCGATCCCACCAC-3’ | Mutant verification |
| P2_rev | 5’-TTCCGGGGATCCGTCGACCT-3’ | Mutant verification |
| kan-2_rev | 5’-ACTCTGGCGCATCGGGCTTC-3’ | Transposon insertion and mutant verification |
| R6Kγori_for | 5'-CCTCTTTCTCCGCACCCGAC-3' | Transposon insertion verification |
| 16S_for | 5’-TGTCGTCAGCTCGTGTCGTGA-3’ | qRT-PCR |
| 16S_rev | 5’-ATCCCCACCTTCCTCCGGT-3’ | qRT-PCR |
| csgD_RT_for | 5’-CCCGTACCGCGACATTG-3’ | qRT-PCR |
| csgD_RT_rev | 5’-ACGTTCTTGATCCTCCATGGA-3’ | qRT-PCR |
| csgB_RT_for | 5’-CATAATTGGTCAAGCTGGGACTAA-3’ | qRT-PCR |
| csgB_RT_rev | 5’-GCAACAACCGCCAAAAGTTT-3’ | qRT-PCR |
| adrA_RT_for | 5’-GGCTGGGTCAGCTACCAG-3’ | qRT-PCR |
| adrA_RT_rev | 5’-CGTCGGTTATACACGCCCG-3’ | qRT-PCR |
| bcsA_RT_for | 5’-GACGCTGGTGGCGCTG-3’ | qRT-PCR |
| bcsA_RT_rev | 5’-GGGCCGCGAGATCACC-3’ | qRT-PCR |
| udp_RT_for | 5’-CGATTTACAAGGGGCTACGC-3’ | qRT-PCR |
| udp_RT_rev | 5’-GTGAATTCGCGGTGAGATGC-3’ | qRT-PCR |
